# Effectiveness of Local Mother's Kitchen Recipe Talks in Reducing the Burden of Anemia Among Children Under Five, Adolescents, Pregnant Women, and Lactating Mothers in Guntur District, India: A Community-Based Intervention Trial

**DOI:** 10.7759/cureus.88215

**Published:** 2025-07-18

**Authors:** Arti Gupta, Vishnu Rajan, Venkatashiva Reddy B, Mounika Kollimarla, Rajeev Aravindakshan

**Affiliations:** 1 Department of Community and Family Medicine, All India Institute of Medical Sciences, Mangalagiri, Mangalagiri, IND

**Keywords:** anemia, community based trial, dietary iron intake, field trial, malnutrition, mother's kitchen

## Abstract

Background

Iron deficiency anemia remains a significant public health concern, particularly among vulnerable groups such as children under five, adolescents, pregnant women, and lactating mothers in low- and middle-income countries. Sustainable, community-driven dietary interventions leveraging locally available foods could provide a culturally acceptable alternative.

Objective

This study assesses the effectiveness of a community-based intervention that incorporates local mother's kitchen recipes and nutrition education talks in enhancing hemoglobin and dietary iron intake levels among targeted groups in the Guntur District, India.

Materials and methods

A one-year community-based intervention trial was conducted in two randomly selected villages, with one village as the intervention arm and the other as the control. A total of 504 participants were enrolled, including children under five, primary school children, and adolescents. The intervention included hands-on cooking sessions that promoted iron-rich, locally available recipes, as well as structured educational talks. Hemoglobin, dietary iron levels, and anthropometric measurements were assessed at baseline and at the post-intervention time point. Data analysis involved difference-in-difference analysis and McNemar tests to assess changes in key parameters.

Results

Participants who attended more than six sessions demonstrated an increase in hemoglobin levels, particularly among children under five (β = 0.70, 95% CI: -0.24 to 1.60) and primary school children (β = 0.40, 95% CI: -0.74 to 1.54). Significant reductions in stunting (92% to 7.7%), underweight, and wasting were observed in children under five. While some reproductive-age women showed declining dietary iron levels, hemoglobin levels improved.

Conclusion

The mother's kitchen model fosters sustainable dietary modifications, improving anemia status and nutritional outcomes beyond pharmacological supplementation.

## Introduction

Iron deficiency anemia (IDA) is the most common dietary disease in the world, affecting people of all ages, genders, and physiological conditions. Preschool children, adolescent girls, pregnant women, and breastfeeding mothers of low- and middle-income countries are among the most vulnerable. In 2019, anemia alone contributed to the 50 million years of life lost due to disability globally, in which IDA is the most common cause [1]. IDA has a negative impact on children's productivity and cognition [2,3], indicating that quick action is needed to address iron deficiency in South Asian underdeveloped countries. In India, despite decades of interventions such as iron-folic acid supplementation programs, IDA remains a persistent challenge, especially in rural and tribal areas [4]. According to a nationally representative survey, 58% of pregnant women and 67% of children under five are anemic [5]. Existing iron supplementation programs face challenges, including low adherence due to side effects, lack of awareness, and logistical barriers in rural settings [6,7]. Moreover, dietary diversity and consumption of iron-rich foods remain suboptimal due to illiteracy, socioeconomic constraints, and cultural practices [8]. This suggests the need for immediate interventions that extend beyond biomedical approaches to more sustainable solutions, engaging communities in their culturally specific contexts.

Evidence on dietary interventions, especially those utilizing locally available and culturally familiar foods, is a valuable tool in combating IDA in a sustainable way [9]. The "mother's kitchen" concept at the local level is designed to train individuals to prepare a rich diet using inexpensive and readily available resources. The local recipes should also contain micronutrients such as vitamin C that enhance iron absorption from the gut. Such local recipes not only address the nutritional needs of the population but also empower the communities by revitalizing their traditional culinary knowledge. Additionally, regular community-based education and discussion sessions will enhance awareness, dispel misconceptions, and foster behavioral change [10]. Therefore, in conjunction with existing pharmacological interventions, this combined approach of utilizing local recipes and community engagement may offer a sustainable solution to combat IDA.

There is a dearth of evidence regarding local kitchen and community education in India. Unlike traditional approaches, which rely solely on pharmacological therapy, the local mother's kitchen emphasizes dietary modification using locally sourced, affordable, and culturally accepted foods. The primary objective of this study is to evaluate the effectiveness of local mother's kitchen and nutrition education talks in reducing IDA prevalence among children under five, adolescents, pregnant women, and lactating mothers in the Guntur District, Andhra Pradesh, India. Additionally, the impact of this intervention on biochemical parameters, such as hemoglobin levels, dietary iron intake, and anthropometric measurements, was also assessed.

## Materials and methods

Study design and duration

A community-based intervention trial was conducted to determine the effectiveness of local mother's kitchen recipes and talks over a one-year period from 2023 to 2024 in rural households of Guntur District, Andhra Pradesh, India. Our study is a field trial without randomization, and the sampling units were selected randomly.

Study setting

The study was conducted in the rural households of Chirravuru and Nidamarru villages located in the Guntur District, Andhra Pradesh, India. The study was conducted at the rural field practice area of the Centre for Rural Health, All India Institute of Medical Sciences, Nuthakki, a rural training center affiliated with a tertiary healthcare institute in the district.

Study population

The study participants included pregnant women in any trimester, lactating women with infants, children under five (aged 6-59 months), primary school children (aged 5-9 years), and adolescents (aged 10-19 years). For adolescents, both school-going and non-school-going individuals were included. All participants were required to have been residing in the Mangalagiri Mandal of Guntur District for at least the past six months. Individuals who were temporarily visiting or living in the area for less than six months, those suffering from serious illness at the time of data collection, or those who were unwilling to provide informed consent (or assent in the case of minors) were excluded from the study. The presence of illness in other household members was not an exclusion criterion unless it posed a direct barrier to data collection or safety concerns.

Sample size and sampling

The sample size calculation assumes two study groups, each receiving a different treatment. The primary endpoint is a continuous variable, hypothesizing that hemoglobin levels in the trial arm will be 11 ± 2 g/dL, with an anticipated 10% increase in mean hemoglobin levels in the intervention arm. Using an alpha level of 0.05, a beta of 0.2, and a power of 0.8, the required sample size for each group is 63 participants. With three Anganwadi centers and one school included in each arm, the total sample size is approximately 250 participants per arm. This study comprises 63 adolescents (both girls and boys), 63 primary school children aged five to nine years, 63 children under five, 10 pregnant women, 20 lactating mothers, and 33 women of reproductive age.

Two villages with nearly equal populations in Mangalagiri Mandal were randomly selected for the study. One village served as the intervention arm, while the other was designated as the control arm. In each village, three Anganwadis and one school were enrolled for the study. To minimize the risk of contamination between the groups, we ensured that participants in the control and intervention groups were selected from separate clusters or locations where possible. Additionally, participants were not informed about the specific components of the intervention to reduce the likelihood of behavioral crossover, such as adopting similar cooking practices.

A list of pregnant women, lactating mothers, adolescents, and children under five associated with the selected Anganwadis was obtained from the Integrated Child Development Services (ICDS) records.

Study procedure

A house-to-house data collection was conducted in the selected villages, including all eligible pregnant women, lactating mothers, adolescents, and non-lactating mothers of children under five. Participants were provided with a patient information sheet detailing the study objectives, procedures, and participants' rights. The study process was explained, and written consent was obtained from participants who were willing to participate. The school principal received the information sheet for adolescents, and written assent was obtained upon agreement to participate. Written informed consent was obtained from pregnant women, lactating women, women of reproductive age, and mothers of children under five.

The participants were interviewed using structured interview tools that included sociodemographic details, anthropometric measurements, and hemoglobin estimation. Three non-consecutive 24-hour dietary recalls were conducted to assess nutrient intake. These assessments were carried out at the participant's home, school, or Anganwadi, preferably outdoors during daylight hours. The anthropometric evaluation was performed using standard methods, including measuring stations and infantometers (seca GmbH & Co. KG, Hamburg, Germany). The tared weighing was conducted for children. Hemoglobin estimation was performed on the spot using hemoglobin devices (HemoCue AB, Ängelholm, Sweden; ACON Laboratories, Inc., San Diego, CA, USA) with capillary blood. The criteria for anemia and malnutrition are included in the supplementary file (Appendices).

In the intervention arm, local recipe talks were organized in a "mother's kitchen" setting. Sessions were conducted weekly for antenatal and postnatal mothers and every 15 days for adolescents. A monthly session is conducted for mothers of children under five at their homes or Anganwadis. Separate sessions were conducted for pregnant women, lactating mothers, adolescents, and mothers of children under five, ensuring each participant actively engaged in the sessions. The mother's kitchen was set up in a participant's home or an Anganwadi. Anganwadi workers, helpers, and school teachers were also encouraged to participate in the discussions. These recipes followed the guidelines of the National Institute of Nutrition, and sessions were audio- or video-recorded with prior consent. The mother kitchen recipe was developed by a team of dieticians and medical experts under the ICMR project number. 4261/2020. The data collection and recipe sessions were conducted by the field staff of the Centre for Rural Health, All India Institute of Medical Sciences, Nuthakki, who had undergone intensive training in preparing the recipe and delivering the sessions. The details of the mother's kitchen recipe have been published elsewhere [11].

This intervention was implemented in addition to ongoing public health initiatives under the Anemia Mukt Bharat program in the trial arm. In contrast, both arms received standard interventions under the same program. At the end of six months, participants in both arms underwent repeat hemoglobin estimation and anthropometric measurements. The primary outcomes of the study were changes in mean hemoglobin and dietary iron levels, while the secondary outcomes included anthropometric parameters. The study is ethically approved by the Institutional Ethics Committee of All India Institute of Medical Sciences, Mangalagiri (approval number: AIIMS/MG/IEC/2022-23/225). The field trial is registered in the Clinical Trials Registry-India (CTRI) under CTRI/2023/02/049591, dated 9th February 2023.

Data analysis

The data analysis will be conducted using R version 4.2.2 (R Foundation for Statistical Computing, Vienna, Austria), WHO Anthro (World Health Organization, Geneva, Switzerland), and DietCalc software (with the new Database from the book - Longvah T, Ananthan R, Bhaskarachary K, Venkaiah K. Indian Food Composition Tables. National Institute of Nutrition, Indian Council of Medical Research, 2017 by Profound Tech solutions, India). Categorical variables are presented as frequencies and percentages, while continuous variables, such as hemoglobin and dietary iron levels, are presented as mean (SD) or median (IQR), depending on their normality. Anthropometric measurements will be analyzed based on the WHO 2006 growth standards using the WHO Anthro software. For estimating iron intake in the diet, DietCalc software was used.

A difference-in-difference analysis was employed to assess changes in mean dietary iron and hemoglobin levels before and after the intervention, stratified by the number of sessions attended by the study participants. Similarly, the McNemar test is used to analyze changes in anthropometric parameters.

## Results

**Figure 1:**
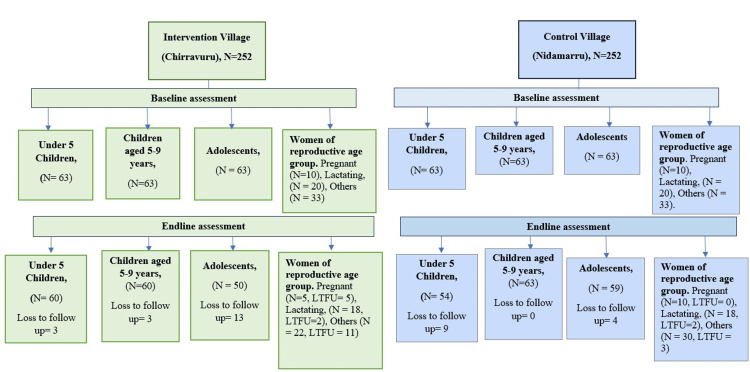
Flowchart of participants throughout the study LTFU: loss to follow-up

A total of 504 participants, including adolescents, children under five, primary school children (6-9 years), pregnant and lactating women, and women of reproductive age, were enrolled, with 63 participants each in the control (Nidamarru) and intervention (Chirravuru) groups across all age categories (Figure 1). The gender distribution was comparable across groups, with females comprising 56-59% of the population in each category. Among adolescents, all participants in the control group had completed primary or high school education, compared to 90% (n = 56) in the intervention group. The socioeconomic status, assessed using the Modified BG Prasad Scale, showed a predominance of Class III households in both groups, with a median per capita income of ₹3,750 (IQR: ₹3,000-₹5,000) (Table 1).

**Table 1:** Baseline sociodemographic and biochemical parameters of children under five, primary school children, and adolescents *chi-squared test, ^#^Mann-Whitney U test, ^$^unpaired t-test, INR: Indian Rupee, IQR: interquartile range, SD: standard deviation

Variable		Nidamarru (control) n (%)	Chirravuru (intervention) n (%)	p-value*	Test statistic
Adolescents					
Gender					
Female		35 (56)	36 (57)	0.9	χ² = 0.01
Male		28 (44)	27 (43)		
Education					
Primary/high school		63 (100)	57 (90)	0.02	χ² = 7.15
Intermediate/diploma		0 (0)	5 (7.9)		
Degree		0 (0)	1 (1.6)		
Father’s education					
Upper primary		36 (61)	32 (54)	0.6	χ² = 2.03
Illiterate/primary		19 (32)	19 (32)		
Intermediate		0 (0)	2 (3.4)		
Degree		4 (6.8)	5 (8.5)		
Post-graduate		0 (0)	1 (1.7)		
Monthly per-capita income in INR, median (IQR)		3,750 (3,000, 5,000)	3,750 (3,000, 5,000)	>0.9^#^	U = 1103
Socioeconomic status (Modified BG Prasad Scale)					
Class IV (1183-2366)		2 (3.2)	5 (7.9)	0.4	χ² = 1.83
Class III (2367-3943)		39 (62)	41 (65)		
Class II (3944-7888)		22 (35)	17 (27)		
Type of house					
Kutcha		1 (1.6)	6 (9.5)	0.06	χ² = 5.53
Pucca		35 (56)	39 (62)		
Semi pucca		27 (43)	18 (29)		
Type of the family					
Extended		21 (33)	14 (22)	0.2	χ² = 1.64
Nuclear		42 (67)	49 (78)		
Weight in Kgs, mean ± SD		37 ± 8	43 ± 14	0.04^$^	t = -2.08
Height in cms, mean ±SD		145 ± 9	149 ± 8	0.008^$^	t = -2.72
Children under five					
Gender	n (%)		n (%)	0.7	χ² = 0.15
Female	37 (59)		35 (56)		
Male	26 (41)		28 (44)		
Father’s education					
Upper primary	20 (32)		27 (45)	0.004	χ² = 13.2
Illiterate/primary	4 (6.3)		6 (10)		
Intermediate	7 (11)		14 (23)		
Degree	21 (33)		12 (20)		
Post-graduate	11 (17)		1 (1.7)		
Monthly per-capita income in INR, median (IQR)	3,750 (3,000, 5,000)		3,750 (3,000, 5,000)	0.8^#^	U = 970
Socioeconomic status (Modified BG Prasad Scale)					
Class IV (1183-2366 INR)	10 (16)		13 (21)	0.4	χ² = 1.82
Class III (2367-3943 INR)	32 (51)		36 (57)		
Class II (3944-7888 INR)	21 (33)		14 (22)		
Type of house					
Kutcha	0 (0)		1 (1.6)	0.2	χ² = 2.82
Pucca	59 (94)		53 (84)		
Semi pucca	4 (6.3)		9 (14)		
Type of the family					
Extended	32 (51)		37 (59)	0.4	χ² = 0.71
Nuclear	31 (49)		26 (41)		
Weight in Kgs, mean ± SD	12.57 ± 3.13		12.58 ± 3.12	0.8^$^	t = -0.25
Height in cms, mean ± SD	89 ± 11		89 ± 10	>0.9^$^	t = 0.02
Primary school children aged 6-9 years					
Gender					
Female	36 (57)		36 (57)	>0.9	χ² = 0.01
Male	27 (43)		27 (43)		
Father’s education					
Upper primary	34 (60)		36 (59)	0.8	χ² = 0.98
Illiterate/primary	11 (19)		14 (23)		
Intermediate	6 (11)		4 (6.6)		
Degree	4 (7.0)		6 (9.8)		
Post-graduate	2 (3.5)		1 (1.6)		
Monthly per-capita income in INR, median (IQR)	3,750 (3,000, 5,000)		3,750 (3,000, 5,000)	0.4^#^	U = 898
Socio-economic status (Modified BG Prasad Scale)					
Class IV (1183-2366 INR)	2 (3.2)		11 (17)	0.03	χ² = 6.84
Class III (2367-3943 INR)	44 (70)		38 (60)		
Class II (3944-7888 INR)	17 (27)		14 (22)		
Type of house					
Kutcha	3 (4.8)		1 (1.6)	0.7	χ² = 0.72
Pucca	44 (70)		47 (75)		
Semi pucca	16 (25)		15 (24)		
Type of the family					
Extended	17 (27)		21 (33)	0.4	χ² = 0.67
Nuclear	46 (73)		42 (67)		
Weight in Kgs, mean ± SD	21.7 ± 5.9		24.1 ± 5.7	0.007^$^	t = -2.76
Height in cms, mean ± SD	122 ± 10		125 ± 11	0.15^$^	t = -1.45

The study included 126 women of reproductive age, equally distributed between the control and intervention groups. Among the participants, 20 (32%) were lactating women, 10 (16%) were pregnant, and 33 (52%) were non-pregnant, non-lactating women. Detailed sociodemographic data are in Table 2.

**Table 2:** Baseline sociodemographic and biochemical parameters of reproductive age women comprising pregnant and lactating women *chi-squared test,^ #^Mann-Whitney U test, ^$^unpaired t-test, INR: Indian Rupee, IQR: interquartile range, SD: standard deviation, ^Fisher's exact test (test-statistics are not available for Fisher's exact test)

Variable	Nidamarru (control) n = 63, n (%)	Chirravuru (intervention) n = 63, n (%)	p-value*	test statistic
Category				
Lactating women	20 (32)	20 (32)	>0.9	χ² = 0.01
Pregnant women	10 (16)	10 (16)		
Women of reproductive group	33 (52)	33 (52)		
Education				
Illiterate	1 (1.6)	6 (9.5)	0.3	χ² = 4.10
Primary/high school	21 (33)	23 (37)		
Intermediate/diploma	20 (32)	20 (32)		
Graduate	18 (29)	13 (21)		
Post-graduate	3 (4.8)	1 (1.6)		
Monthly per-capita income in INR, median (IQR)	5,000 (3,333, 5,000)	5,000 (3,571, 6,250)	0.2^#^	U = 1608
Socioeconomic status (Modified BG Prasad Scale)				
Class IV (1183-2366 INR)	10 (16)	18 (29)	0.02	χ² = 9.65
Class III (2367-3943 INR)	24 (38)	28 (44)		
Class II (3944-7888 INR)	29 (46)	15 (24)		
Class I (7889 and above INR)	0 (0)	2 (3.2)		
Type of house				
Kutcha	0 (0)	1 (1.6)	0.04	χ² = 6.46
Pucca	60 (95)	52 (83)		
Semi pucca	3 (4.8)	10 (16)		
Type of the family				
Extended	31 (49)	32 (51)	0.9	χ² = 0.01
Nuclear	32 (51)	31 (49)		
Marital status				
Married	59 (97)	59 (94)	0.7	χ² = 0.15
Unmarried	2 (3.3)	4 (6.3)		
Age at marriage (in years)				
≤18	22 (37)	29 (51)	0.14	χ² = 2.20
>19	37 (63)	28 (49)		
Consanguinous marriage				
Yes	24 (41)	17 (28)	0.2	χ² = 1.77
No	35 (59)	43 (72)		
Lactating				
Yes	21 (38)	20 (40)	0.8	χ² = 0.06
No	34 (62)	30 (60)		
Gravida				
1	11 (20)	8 (16)	0.6	χ² = 2.70
2	30 (55)	32 (64)		
3	9 (16)	8 (16)		
4	2 (3.6)	2 (4.0)		
5	3 (5.5)	0 (0)		
Para				
0	0 (0)	1 (2.0)	0.9^	
1	17 (31)	12 (24)		
2	32 (58)	31 (63)		
3	5 (9.1)	5 (10)		
5	1 (1.8)	0 (0)		
Abortion				
0	45 (82)	41 (84)	0.8	χ² = 0.44
1	7 (13)	7 (14)		
2	1 (1.8)	1 (2.0)		
3	2 (3.6)	0 (0)		
Still birth	1 (1.8)	4 (8.0)	0.2	χ² = 1.71
No. of live children				
0	0 (0)	2 (4.0)	0.7^	
1	18 (33)	13 (26)		
2	31 (56)	31 (62)		
3	5 (9.1)	4 (8.0)		
5	1 (1.8)	0 (0)		
Weight in Kgs, mean ± SD	59 ± 13	59 ± 10	0.7^$^	t = 0.39
Height in cms, mean ± SD	152 ± 6	153 ± 6	0.5^$^	t = -0.67

Baseline hemoglobin and dietary iron intake of participants are presented in Table 3. Among adolescents, mean hemoglobin levels were not significantly different in the control and intervention groups (10.89 g/dL vs. 11.03 g/dL, p = 0.9), with moderate anemia being more prevalent in the intervention group (n = 32, 51% vs. n = 26, 41%). Children under five in the intervention group had lower mean hemoglobin (9.36 g/dL vs. 10.31 g/dL). Primary school children in the intervention group also showed lower mean hemoglobin levels and a higher prevalence of moderate anemia. Pregnant women in the intervention group had a lower mean hemoglobin level (9.66 g/dL vs. 10.21 g/dL), with a higher prevalence of moderate anemia (n = 6, 60% vs. n = 3, 30%), but higher dietary iron levels. Lactating and reproductive-age women in the intervention group had lower mean hemoglobin levels and a higher prevalence of moderate anemia (n = 28, 53% vs. n = 22, 42%). Dietary iron levels were slightly higher in the intervention group across most categories, except for children under five, where levels were comparable.

**Table 3:** Baseline hemoglobin and dietary iron levels of participants in the control and intervention groups *chi-squared test, ^$^unpaired t-test, ^Fisher's exact (test-statistics are not available for Fisher's exact test), SD: standard deviation

Variable		Nidamarru (control) n = 63, n (%)	Chirravuru (intervention) n = 63, n (%)	p-value*	Test statistic
Adolescents					
Hemoglobin, mean ± SD		10.89 ± 1.27	11.03 ± 1.06	0.9^$^	t = -0.13
Anemia severity					
Mild anemia		26 (41)	19 (30)	0.6^	
Moderate anemia		26 (41)	32 (51)		
Severe anemia		2 (3.2)	0 (0)		
No anemia		9 (14)	12 (19)		
Dietary iron, mean ± SD		4.03 ± 2.98	4.58 ± 3.53	0.12^$^	t = -1.57
Children under five					
Hemoglobin, mean ± SD	10.31 ± 1.22		9.36 ± 1.46	<0.001^$^	t = 3.94
Severity of anemia					
Mild anemia	22 (35)		13 (21)	<0.001^	
Moderate anemia	21 (33)		36 (57)		
Severe anemia	0 (0)		6 (9.5)		
No anemia	20 (32)		8 (13)		
Dietary iron, mean ± SD	2.01 ± 1.40		1.95 ± 1.35	0.6^$^	t = 0.53
Primary school children					
Hemoglobin, mean ± SD	11.13 ± 0.92		10.68 ± 1.00	0.009^$^	t = 2.67
Severity of anemia					
Mild anemia	8 (13)		16 (25)	0.002^	
Moderate anemia	27 (43)		36 (57)		
Severe anemia	0 (0)		1 (1.6)		
No anemia	28 (44)		10 (16)		
Dietary iron, mean ± SD	4.18 ± 2.73		4.45 ± 3.35	0.3^$^	t = -1.04
Pregnant women					
Hemoglobin, mean ± SD	10.21 ± 0.79		9.66 ± 1.33	0.3^$^	t = 1.06
Mild anemia	6 (60%)		2 (20%)	0.3^	
Moderate anemia	3 (30%)		6 (60%)		
No anemia	1 (10%)		2 (20%)		
Dietary iron, mean ± SD	4.48 ± 1.17		8.47 ± 5.49	0.001^$^	t = -4.12
Lactating and reproductive age women					
Hemoglobin, mean ± SD	10.86 ± 1.47		10.36 ± 1.58	0.10^$^	t = 1.68
Severity of anemia					
Mild anemia	14 (26%)		14 (26%)	0.2	χ² = 4.55
Moderate anemia	22 (42%)		28 (53%)		
No anemia	15 (28%)		7 (13%)		
Severe anemia	2 (3.8%)		4 (7.5%		
Dietary iron, mean ± SD	4.64 ±1.78		4.95 ± 1.96	0.3^$^	t = -1.01

The anthropometric characteristics of participants across intervention and control groups are summarized in Table 4. Anthropometric characteristics showed severe stunting (n = 14, 22% vs. n = 9, 14%) in the control group and severe underweight (n = 14, 22% vs. n = 3, 4.8%) among children under five in the intervention group. Wasting was similar, but severe wasting was higher in the intervention group (n = 8, 13% vs. n = 2, 3.2%). In primary school children, a normal BMI was slightly more prevalent in the intervention group (n=44, 70% vs. n=41, 65%), while severe thinness was lower (3.2% vs. 13%). Among adolescents, obesity was more prevalent in the intervention group (n = 7, 11%) compared to the control group (n = 1, 1.6%). For women of reproductive age, obesity rates were identical (n = 27, 51%), but underweight rates were higher in the intervention group (n = 5, 9.4% vs. n = 3, 5.7%).

**Table 4:** Baseline anthropometric characteristics of participants in the intervention and control groups *chi-squared test, BMI: body mass index (kg/m^2^)

Group	Characteristic	Nidamarru (control) n (%)	Chirravuru (intervention) n (%)	p-value*	Test statistic
Children under five	Stunting	n = 63	n = 63	<0.001	χ² = 22.19
	Normal	44 (70)	20 (32)		
	Severe stunting	9 (14)	14 (22)		
	Stunting	10 (16)	29 (46)		
	Underweight				
	Normal	54 (86)	39 (62)	0.005	χ² = 10.61
	Severe underweight	3 (4.8)	14 (22)		
	Underweight	6 (9.5)	10 (16)		
	Wasting				
	Normal	56 (89)	50 (79)	0.14	χ² = 3.93
	Wasted	5 (7.9)	5 (7.9)		
	Severely wasted	2 (3.2)	8 (13)		
Primary school children	BMI for age	n = 63	n = 63	0.2	χ² = 4.56
	Normal	41 (65)	44 (70)		
	Overweight	5 (7.9)	8 (13)		
	Thin	9 (14)	9 (14)		
	Severe thin	8 (13)	2 (3.2)		
Adolescents	BMI for age	n = 63	n = 63	0.2	χ² = 5.37
	Normal	43 (68)	34 (54)		
	Obesity	1 (1.6)	7 (11)		
	Overweight	10 (16)	11 (17)		
	Thin	8 (13)	8 (13)		
	Severe thin	1 (1.6)	3 (4.8)		
Women of reproductive age (excluding pregnant women)	BMI	n = 53	n = 53	0.7	χ² = 1.38
	Normal	16 (30)	12 (23)		
	Obese	27 (51)	27 (51)		
	Overweight	7 (13)	9 (17)		
	Underweight	3 (5.7)	5 (9.4)		

**Figure 2:**
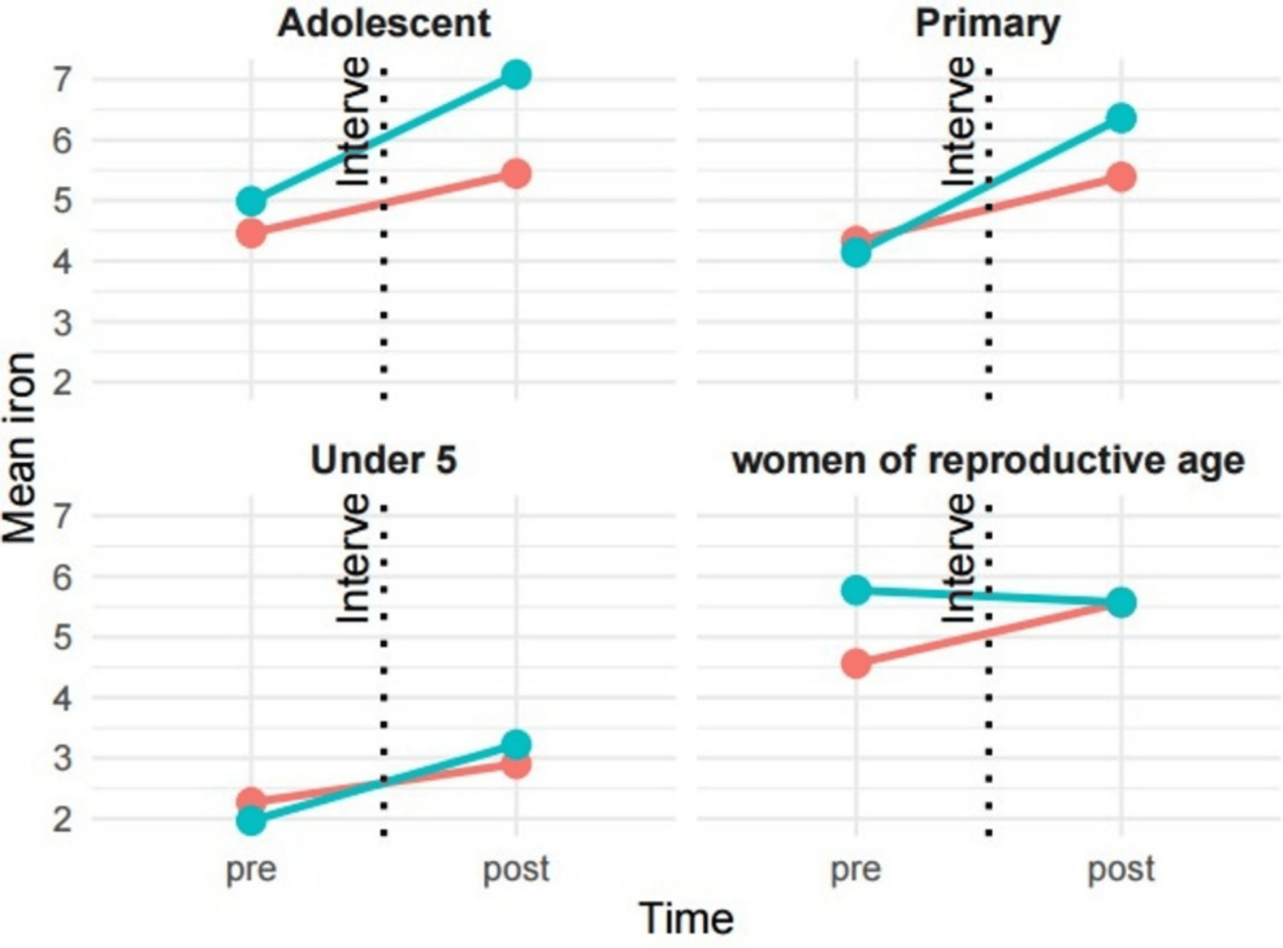
Change in mean dietary iron intake levels of participants stratified by the number of sessions attended Red: ≤5 visits, blue: ≥6 visits

Differences-in-differences analysis has shown the change in mean dietary iron and hemoglobin levels from pre- to post-intervention, according to the number of sessions attended by the study participants. Adolescents who attended more than six sessions experienced a 1.1-unit increase in dietary iron intake (β = 1.1, 95% CI: -0.16 to 2.4) compared to those with fewer sessions. Similarly, primary school children who attended more than six sessions showed a 1.2-unit increase in dietary iron levels (β = 1.2, 95% CI: -1.1 to 3.4) compared to their peers who attended five sessions or fewer. In contrast, a declining trend in dietary iron levels was observed among reproductive-age women who attended more than five sessions (Figure 2).

**Figure 3:**
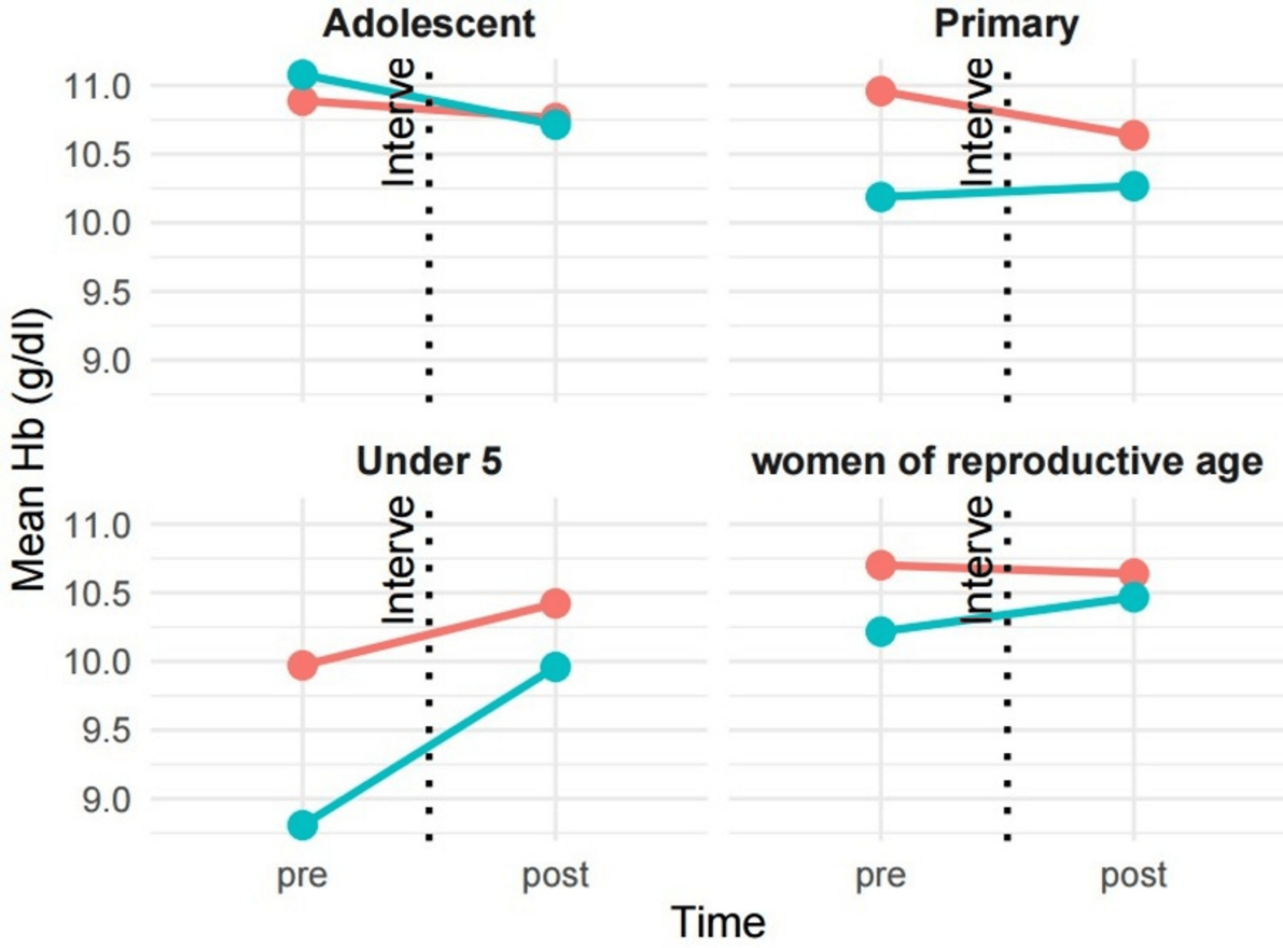
Change in mean hemoglobin levels of participants stratified by the number of sessions attended Red: ≤5 visits, blue: ≥6 visits

However, despite a declining trend in serum iron levels, hemoglobin levels of reproductive-age women increased by 0.31 units (β = 0.31, 95% CI: -0.41 to 1.0) compared to those who attended fewer sessions. Similarly, there is an increase in hemoglobin levels in children under five and primary school children who had participated in more than six sessions. Conversely, there is a decrease in hemoglobin levels among adolescents who had attended more than six sessions, though it is not statistically significant (Figure 3).

Among children under five, stunting improved in the ≤5 visits group (from 83% to 17%), while a substantial decline occurred in the ≥6 visits group (from 92% to 7.7%). The proportion of underweight and wasted individuals declined significantly in both groups (Table 5).

**Table 5:** Anthropometric changes in children under under based on the frequency of intervention sessions attended

	≤5 visits				≥6 visits			
	Pre n = 111 n (%)	Post n = 99 n (%)	p-value (McNemar)	Test statistic	Pre n = 15, n (%)	Post n = 15, n (%)	p-value (McNemar)	Test statistic
Stunting status								
No stunting	58 (40)	88 (60)	0.004	χ² = 8.29	3 (18)	14 (82)	0.84	χ² = 0.04
Stunting	53 (83)	11 (17)			12 (92)	1 (7.7)		
Underweight status								
No underweight	81 (48)	89 (52)	<0.001	χ² = 14.76	10 (45)	12 (55)	0.14	χ² = 2.18
Underweight	30 (75)	10 (25)			5 (63)	3 (38)		
Wasting status								
No wasting	93 (52)	85 (48)	<0.001	χ² = 13.15	12 (48)	13 (52)	0.02	χ² = 5.33
Wasting	18 (56)	14 (44)			3 (60)	2 (40)		

## Discussion

The findings of this community-based intervention trial highlight the effectiveness of a locally tailored dietary and educational approach in reducing the burden of IDA among vulnerable populations, including children under five, adolescents, pregnant women, and lactating mothers in the Guntur District.

Prior studies have emphasized the importance of dietary interventions in addressing micronutrient deficiencies, particularly among populations with limited access to fortified foods and supplements [12,13]. Additionally, incorporating iron absorption enhancers, such as vitamin C-rich foods, further optimizes iron bioavailability, aligning with findings from earlier nutritional research [14].

Changes in mean hemoglobin and dietary iron levels

The improvement in hemoglobin levels and reduction in anemia prevalence among participants who attended more than six sessions underscore the role of dietary interventions in combating IDA. Despite the positive trend in hemoglobin levels post-intervention, statistical significance was not achieved. The relatively short duration of the intervention may not have been sufficient to elicit a statistically significant change in hemoglobin levels despite observable clinical improvements. Pregnant and lactating women benefited from the intervention, with improvements in mean hemoglobin and dietary iron among those who had attended more than six sessions. This finding is consistent with previous literature, which indicates that food-based interventions are more sustainable and acceptable in rural settings [15]. A similar intervention, conducted in a local mother's kitchen for tribal pregnant and lactating women in Andhra Pradesh, showed increased dietary iron intake and higher hemoglobin levels among those who attended more than 10 sessions [16].

There is a dearth of studies on the impact of local mother's kitchen recipes, particularly on children under five, primary school children, and adolescents. Our study has shown that the local mother's kitchen recipe effectively improves anemia parameters among these groups. Although the increase was greater among those who attended more than six sessions, even participants with fewer sessions showed improvement. This can be attributed to the country's ongoing efforts to eliminate anemia through the Anemia Mukt Bharat Program [17]. The emphasis on locally available, affordable, and culturally accepted iron-rich foods improved compliance compared to pharmacological supplementation alone.

The declining trend in dietary iron levels among some reproductive-age women despite an increase in hemoglobin suggests that additional factors, such as chronic inflammation or parasitic infections, may influence iron metabolism and require further investigation. Similarly, a negative trend was noted among adolescents who attended more than six sessions. Adolescents may not be directly involved in kitchen activities and cooking, whereas for children under five and those in primary school, the intervention is targeted at the mother. This could be why a positive change is not observed among adolescents. Weekly iron and folic acid supplementation, accompanied by counseling, has been demonstrated to improve anemia among adolescents in studies conducted in India [18,19]. In a large-scale study conducted in Uttar Pradesh, the prevalence of anemia among adolescents decreased from 73.3% to 25.4% over a four-year period. The relatively short duration of the end-line evaluation in the current study may also explain the absence of significant improvement observed [18].

Changes in malnutrition-related parameters

Among children under five, the intervention resulted in a marked decline in stunting, underweight, and wasting, particularly among those who attended more than six intervention sessions. This aligns with evidence suggesting that iron deficiency in early childhood is associated with growth retardation and cognitive impairments, which can be mitigated through improved dietary practices and maternal education [20]. A similar trend was observed among primary school children, where a reduction in thinness and improvement in overall nutritional status were noted, supporting previous studies that emphasize the link between iron status and cognitive performance in school-aged children [21]. The impact of the intervention among adolescents was particularly noteworthy, as this age group exhibited significant reductions in obesity, particularly among those attending more than six sessions. Studies conducted in developed countries have shown that teaching cooking skills leads to improvements in dietary habits and positive changes in eating practices [22,23]. In India, mobile kitchen initiatives, such as "Bhavishya Shakthi," a public health intervention, have successfully educated, empowered, and upskilled marginalized women in Kolkata. These programs have demonstrated improvements in nutrition and health awareness, resulting in healthier dietary practices [24]. The improvements observed in both groups, regardless of the number of sessions attended, can be attributed to several factors. An important factor is the broader impact of national nutritional health programs, particularly Poshan 2.0, the Anaemia Mukt Bharat Program, the Mid-day Meal Program, and the ICDS, which may have contributed to the overall growth outcomes in children under five, primary school children, and adolescents. Additionally, even a few sessions of nutritional education and intervention may have been sufficient to create awareness among participants and their families about the importance of balanced meals.

One of the key strengths of this study was the integration of dietary education with practical cooking demonstrations, ensuring that knowledge translated into behavior change. Our study is the first to assess the effectiveness of local kitchen recipes on a large scale across multiple age groups. Unlike conventional supplementation programs, which often face adherence challenges, the hands-on approach in a familiar environment enabled participants to actively engage and incorporate the recommended dietary modifications into their daily routines. The concept of the mother's kitchen, where community members were trained to prepare iron-rich meals using locally available resources, had a profoundly positive impact on the community's nutritional status. The observed improvement in hemoglobin levels and dietary behavior can be reasonably attributed to the mother's kitchen recipe talks, given the structured nature of the intervention, the targeted messaging on iron-rich food preparation, and the improvement seen within the intervention group compared to baseline. Since the intervention focused on behavior change communication tailored to local dietary practices, these sessions likely played a direct role in influencing mothers' cooking habits and, subsequently, the family's nutritional intake. However, seasonal variation in food availability, concurrent nutrition-related programs, or external community influences could have contributed to the change. A cluster RCT in Maharashtra involving participatory learning and action groups and cooking demonstrations led to significant improvements in maternal and child dietary practices [25]. Despite existing government programs such as the Weekly Iron and Folic Acid Supplementation program, adherence remains a challenge due to side effects and misconceptions [26]. The community-based approach adopted in this study successfully addressed these barriers by fostering awareness and acceptance of iron-rich dietary modifications.

Despite these promising results, some limitations must be acknowledged. The study was conducted in a specific geographical region, and findings may not be generalizable to other areas with different dietary habits and socio-cultural contexts. Additionally, while the intervention successfully improved hemoglobin levels, long-term sustainability and retention of nutritional practices beyond the study period require further evaluation. The short duration of follow-up may limit the ability to observe longer-term trends or causal relationships, particularly in outcomes that require extended observation to capture meaningful changes. Additionally, the brief period may not fully account for seasonal variations in dietary intake and food availability. Future research should investigate strategies to institutionalize community-based nutrition programs within existing public health frameworks, thereby ensuring sustained benefits for vulnerable populations.

## Conclusions

The local mother's kitchen approach and community-based educational sessions proved to be an effective and culturally relevant strategy in addressing IDA among children under five, adolescents, pregnant women, and lactating mothers. The study highlights the importance of utilizing locally available resources and promoting community participation to achieve sustainable improvements in anemia and overall nutritional status. This can be achieved by integrating community-led cooking demonstrations featuring regionally available iron-rich foods into routine activities at Anganwadi centers. Scaling the "mother's kitchen" model appears feasible through existing government systems such as Anganwadi centers and self-help groups, given their widespread community presence, established infrastructure, and alignment with nutrition-focused initiatives. Despite the positive changes, the sustainability and long-term impact of such interventions still require further research.

## Appendices

**Table 6:** BMI for age for the 5–19 years age group: interpretation of cut-offs Source: World Health Organization (WHO). Growth reference 5–19 years: BMI for age (5–19 years). Available at https://www.who.int/tools/growth-reference-data-for-5to19-years

Category	BMI for age Z-score (SD)	Equivalent BMI at 19 years
Obesity	> +2 SD	BMI > 30 kg/m²
Overweight	> +1 SD to ≤ +2 SD	BMI > 25 kg/m² to ≤ 30 kg/m²
Normal weight	> -2 SD to ≤ +1 SD	-
Thinness	< -2 SD to ≥ -3 SD	-
Severe thinness	< -3 SD	-

**Table 7:** Cut-off values for hemoglobin for IDA IDA: iron deficiency anemia Source: World Health Organization. Haemoglobin concentrations for the diagnosis of anaemia and assessment of severity. Geneva: WHO; 2011. Available from: https://www.who.int/publications/i/item/WHO-NMH-NHD-MNM-11.1

Age groups	No anemia	Mild anemia	Moderate anemia	Severe anemia
Children 6-59 months of age	≥11 g/dL	10-10.9 g/dL	7-9.9 g/dL	<7 g/dL
Children 5-11 years of age	≥11.5 g/dL	11-11.4 g/dL	8-10.9 g/dL	<8 g/dL
Children 12-14 years of age	≥12 g/dL	11-11.9 g/dL	8-10.9 g/dL	<8 g/dL
Non-pregnant women (15 years of age and above)	≥12 g/dL	11-11.9 g/dL	8-10.9 g/dL	<8 g/dL
Pregnant women	≥11 g/dL	10-10.9 g/dL	7-9.9 g/dL	<7 g/dL
Men	≥13 g/dL	11-12.9 g/dL	8-10.9 g/dL	<8 g/dL

**Table 8:** Classification of malnutrition as per BMI for adults Source: World Health Organization. Obesity and overweight. WHO Fact Sheet. Updated June 2021. Available from: https://www.who.int/news-room/fact-sheets/detail/obesity-and-overweight BMI: body mass index

Sr. No.	BMI (kg/m^2^)	Category
1	< 16.0	Severe
2	16.0-16.9	Moderate
3	17.0-18.4	At risk
4	18.5-24.9	Normal
5	25.0-29.9	Overweight
6	> 30	Obese

**Table 9:** Anthropometric parameters for assessing malnutrition in child as per the new WHO standards of malnutrition WFA: weight for age, HFA: height for age, WFH: weight for height, BMI: body mass index Source: WHO Child Growth Standards: Length/height-for-age, weight-for-age, weight-for-length, weight-for-height and body mass index-for-age: Methods and development. Geneva: World Health Organization; 2006. Available from: https://www.who.int/publications/i/item/924154693X

Category	Criteria	Classification	Z score
Underweight	z-scores (SD) below median WFA	Moderate	-3
		Severe	z-score < -3
Stunting	z-scores (SD) below median HFA	Moderate	-3 < z-score < -2
		Severe	z-score < -3
Wasting	z-scores (SD) below median WFH	Moderate	-3 < z-score < -2
		Severe	z-score < -3
